# Ocular Counter Rolling in Astronauts After Short- and Long-Duration Spaceflight

**DOI:** 10.1038/s41598-018-26159-0

**Published:** 2018-05-17

**Authors:** Millard F. Reschke, Scott J. Wood, Gilles Clément

**Affiliations:** 1 NASA, Neuroscience Laboratory, Johnson Space Center, Houston Texas, USA; 2 Lyon Neuroscience Research Center, Bron, France; 3 KBRwyle, Houston, Texas USA

## Abstract

Ocular counter-rolling (OCR) is a reflex generated by the activation of the gravity sensors in the inner ear that stabilizes gaze and posture during head tilt. We compared the OCR measures that were obtained in 6 astronauts before, during, and after a spaceflight lasting 4–6 days with the OCR measures obtained from 6 astronauts before and after a spaceflight lasting 4–9 months. OCR in the short-duration fliers was measured using the afterimage method during head tilt at 15°, 30°, and 45°. OCR in the long-duration fliers was measured using video-oculography during whole body tilt at 25°. A control group of 7 subjects was used to compare OCR measures during head tilt and whole body tilt. No OCR occurred during head tilt in microgravity, and the response returned to normal within 2 hours of return from short-duration spaceflight. However, the amplitude of OCR was reduced for several days after return from long-duration spaceflight. This decrease in amplitude was not accompanied by changes in the asymmetry of OCR between right and left head tilt. These results indicate that the adaptation  of otolith-driven reflexes to microgravity is a long-duration process.

## Introduction

The ability to sense gravity is fundamental for our balance and our spatial orientation. Vertebrates sense gravity using the otolith organs located in the labyrinth inner ear. The sensors consist of calcium carbonate crystals (otoconia) within a gelatinous matrix attached to hair cells (macula). The otoconia move and deform the hair cells when the body inclines or accelerates. The otolith organs are therefore sensitive to both the direction and amplitude of the gravity vector^1^. As a consequence, they cannot discriminate between tilt and translation, which has important physiological implications^2,3^.

On Earth, during static head tilt to the side, the eyes rotate around the line of sight in the direction opposite to the head tilt. This ocular counter-rolling (OCR) reflex allows the horizontal meridian of the retina to remain parallel to the horizon during head roll. Under constant *g* on Earth, OCR is proportional to the sine of the angle of head roll tilt relative to the vertical because the otolith organs sense the acceleration exerted in the plane of the macula, the magnitude of which varies with the sine of the tilt angle^1^. For example, static roll tilts of the head at 15°, 25°, 30°, and 45° are equivalent to linear accelerations in the plane of the utricular macula of 0.26 g, 0.42 g, 0.5 g, and 0.71 g, respectively. The resulting OCR ranges from 3° to 7°, which corresponds to a gain of 0.15–0.2^1^.

In weightlessness, the otolith organs no longer sense the direction of gravity; however, they continue to sense linear accelerations of the body along all three axes. It has been proposed that after adaptation to weightlessness, the central nervous system interprets all signals from the otolith organs as being caused by translation, not tilt^2,3^, which led to the hypothesis that static OCR would be reduced after spaceflight. However, findings are inconsistent^4–11^: studies report OCR decreases after spaceflight in some subjects, and OCR increases or no changes in other subjects. The inconsistency in these results may be due to the small number of crewmembers who were studied, the duration of weightlessness exposure, or the methods used, i.e. whole body tilt, head tilt, or eccentric rotation.

The present study had three objectives: (a) use a normative population on Earth to compare the OCR in response to whole body tilt with the OCR in response to a head tilt relative to the body; (b) compare the OCR in response to head tilt relative to the body in astronauts before, during, and immediately after a short-duration spaceflight; and (c) compare the OCR in response to whole body tilt in astronauts before and immediately after a long-duration space flight.

## Methods

### Participants

Twelve astronauts (10 male, 2 female) aged from 38 to 58 (mean 46.3 years) participated in these studies. OCR was measured in 6 astronauts before, during and after Space Shuttle missions lasting 4–6 days (mean 4.9 days). Six other astronauts were tested before and after International Space Station (ISS) missions lasting 113–286 days (mean 165.8 days).

Space Shuttle astronauts were tested 3 separate times before their mission, at about 2 months, 1 month, and 10 days before the flight. Measurements were also obtained on flight day 1 (FD1), and on FD3 and FD4. OCR was measured within 2 hours of landing (R + 0), and then 3 days later (R + 3). ISS astronauts were measured 6 months, 4 months, and 2 months before the flight. Postflight measurements were made 1 day after landing (R + 1), and then 4 days (R + 4) and 8 days later (R + 8).

Seven non-astronaut subjects (5 male, 2 female) aged from 30 to 45 (mean 38.0 years) participated as a normative population from which measurements of OCR during whole body tilt and head tilt were compared on Earth.

The astronauts passed an Air Force Class II physical, and the normative subjects passed an Air Force Class III physical. Both the astronauts and the normative subjects had no known vestibular, sensory-motor, or other central nervous system complications. All participants had normal 20/20 or better vision without correction, and none used any drugs, including alcohol or anti-motion sickness medications, within 72 hours of a scheduled data collection session.

The experiment was undertaken with the informed and written consent of each subject for both study participation and publication of image in an online open-access publication. The test procedures were approved by the NASA Johnson Space Center Institutional Review Board and were performed in accordance with the ethical standards laid down in the 1964 Declaration of Helsinki.

### Measurements of OCR before, during, and after Space Shuttle Missions

**Figure 1:**
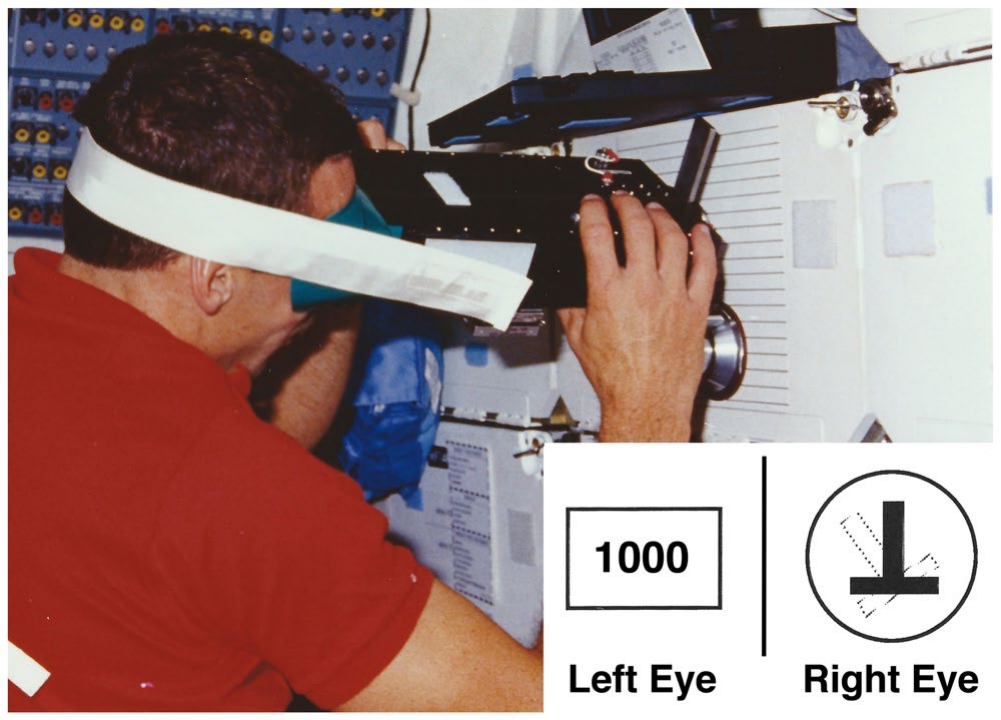
Goggles used to measure OCR on board the Space Shuttle. With his head stabilized to the goggles by a dental bite, the astronaut tilted his head relative to his body. He then viewed an inverted “T” that appeared as an afterimage on the retina (see insert). With his head back in the vertical position (aligned with his body), he matched the retinal afterimage with a reference target using a thumb-wheel. The display was set to an arbitrary unit of 1000. Photo courtesy of NASA.

OCR was measured using afterimages produced by a set of goggles that housed a reference target and a strobe that placed an upside down “T” afterimage on the retina. Looking into the goggles the subject saw with the left eye a digital display that indicated the amount of target rotation (Fig. 1). The inverted “T” served as both the target and reference template and was milled into a disk controlled with a thumb-wheel that allowed the subject to rotate the reference target. Behind the disk was an electronic photographic flash with the flash-ready light positioned so that the inverted “T” was visible to the subject’s right eye. The subject used a thumb-wheel to rotate the reference target until it matched the afterimage. The thumb-wheel and the potentiometer used for the measurement of OCR had slight hysteresis, which resulted in an absolute OCR measurement accuracy of approximately 0.1°.

The goggles were attached to a wall (preflight and post-flight measurements) and to the forward middeck lockers of the Space Shuttle (in-flight measurements) (Fig. 1). On the ground, all data were collected while the crewmembers stood. During flight, the crewmembers’ feet were held to the floor with foot restraints. Both a head strap and custom bite were used to insure that the head was coupled to the goggles. OCR measurements were obtained during static head tilts in the roll plane at angles of 0°, ±15°, ±30°, and ±45°. The order of tilt angles and directions were randomized with the constraint that the same tilt angle would not be used consecutively. Three or more measurements were obtained at each angle of head tilt.

While the subjects’ head was aligned with their body, they were asked to adjust the reference target to the vertical position using the thumb-wheel until a read-out of 1000 was visible on the digital display. Once the reference target was in the vertical position, the crewmembers tilted the goggles and their head to the randomly selected angle of tilt with the assistance of the test operator. The crewmember maintained this position for 30 s to allow the eye torsion to stabilize; the electronic flash was enabled to place the afterimage on the retina, and the subject’s head and the goggles were then returned to the upright position. Once in the upright position, the crewmember shut his eyes, moved the thumb-wheel slightly to the right or left, then opened his eyes and moved the thumb-wheel until the reference target matched the angle of retinal afterimage. The angle of tilt of the reference target was displayed digitally, and was recorded with a voice-activated tape recorder (during flight), or noted in a prepared checklist (before flight and after flight)^12^.

On the ground the goggles were calibrated with a precision carpenter level, and before each data collection the level was used to set the body of the goggles perpendicular to gravity, ensuring that the long arm of the inverted “T” was parallel to gravity when the digital display was set to 1000 (arbitrary unit). During flight the goggles were aligned with a template that was attached to the forward locker and placed such that the head would be aligned with the body (Fig. 1).

### Measurements of OCR before and after ISS missions

**Figure 2:**
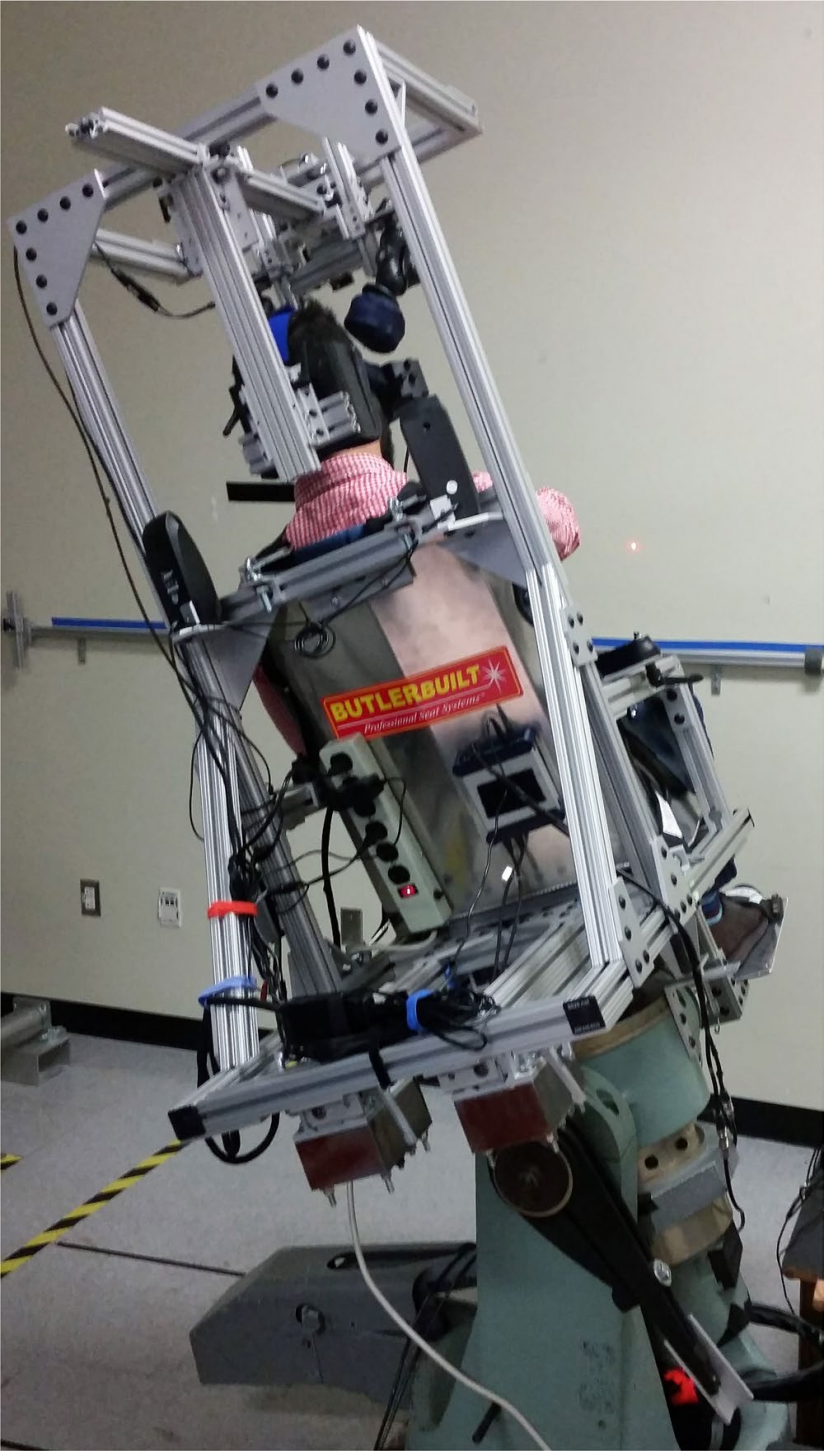
The NASA Johnson Space Center Off-Vertical Axis Rotation chair was used to tilt the ISS crewmembers 25° to the right or to the left.

The crewmembers were seated in the NASA JSC OVAR chair with straps and padding at mid-torso, waist, thighs, and feet. An electromechanical linear actuator tilted the chair and subject off vertical to ±25° orientations (Fig. 2). Moldable Vac-Pacs provided support that helped immobilize the body and distribute the pressure uniformly during body tilt. The chair remained in each tilted position for 3 min in the dark while crewmembers stared straight ahead. Eye movements were recorded using a lightweight binocular video-oculography mask (SensoMotoric Instruments,Teltow, Germany) that was secured to the subject’s head with a Velcro strap. Near-infrared emitting diodes were mounted to the mask to allow the eyes to be recorded in darkness. Eye data were digitally recorded and processed using an eye tracking system described previously^13^. The eye image that was recorded while the subject was in the upright condition was used as a reference; in the tilt positions, the rotatory displacement of the iris landmarks around the center of the pupil was computed as a shift in torsional eye position. A previous study indicated that the accuracy of the OCR measures with this method was <0.1° within the range of ±5° ^13^.

### Measurements of OCR in the normative population

In addition to the OCR investigations in astronauts, normative responses were obtained from 7 subjects during whole body tilt and during upright stance while the head was tilted. The whole body tilt condition was a control experiment to investigate the extent of OCR elicited under normal terrestrial conditions without cervical input and using the afterimage method. The control subjects were tested using the same goggles and the same standing position methods as those used to collect pre- and post-flight data from the Space Shuttle astronauts.

In the whole body tilt condition the NASA JSC Pitch and Roll Device (PARD) was used to roll tilt the body and head together at the same angles of tilt that were used in the standing experiment. The goggles were attached to a pallet directly in front of the subject’s face. With the subject in the vertical position, the reference target was set so that the long arm of the inverted “T” was vertical (and the digital display set to 1000) and aligned with the subject’s head and trunk. The subject and goggles were then moved to a predetermined and randomized angle of tilt by servo control at a rate less than 2°/s^2^, and subject remained in the desired tilt position for 30 s before the afterimage was placed on the retina. The subject then closed his eyes and was immediately returned to the upright position at less than 2°/s^2^. The operator randomly displaced the thumb-wheel, and the upright subject opened his eyes and used the thumb-wheel to rotate the reference target until it matched the retinal afterimage.

### Data analysis

In the 3 studies described above (Space Shuttle, ISS, normative), 3 or more measurements of OCR were obtained for each angle of tilt. For all the tests performed on the ground (astronauts, normative population), the OCR amplitudes of each of the subjects’ 3 trials were averaged for each of the body or head tilt angles. An asymmetry ratio was calculated to compare OCR during right and left tilts:

$${\rm{Asymmetry}}\,{\rm{ratio}}=|({\rm{OCR}}\,{\rm{right}}-{\rm{OCR}}\,{\rm{left}})/({\rm{OCR}}\,{\rm{right}}+{\rm{OCR}}\,{\rm{left}})|\times 100$$

Analyses of variance (ANOVAs) were used to compare values of OCR amplitude and asymmetry after whole body tilt and head tilt, and to compare values during sessions (preflight, in-flight, post-flight). Tests of distribution and homogeneity of variances were also performed to check the normality assumption for ANOVAs.

### Data availability

All data generated or analyzed during this study are included in this article.

## Results

### Ground-based study

**Figure 3:**
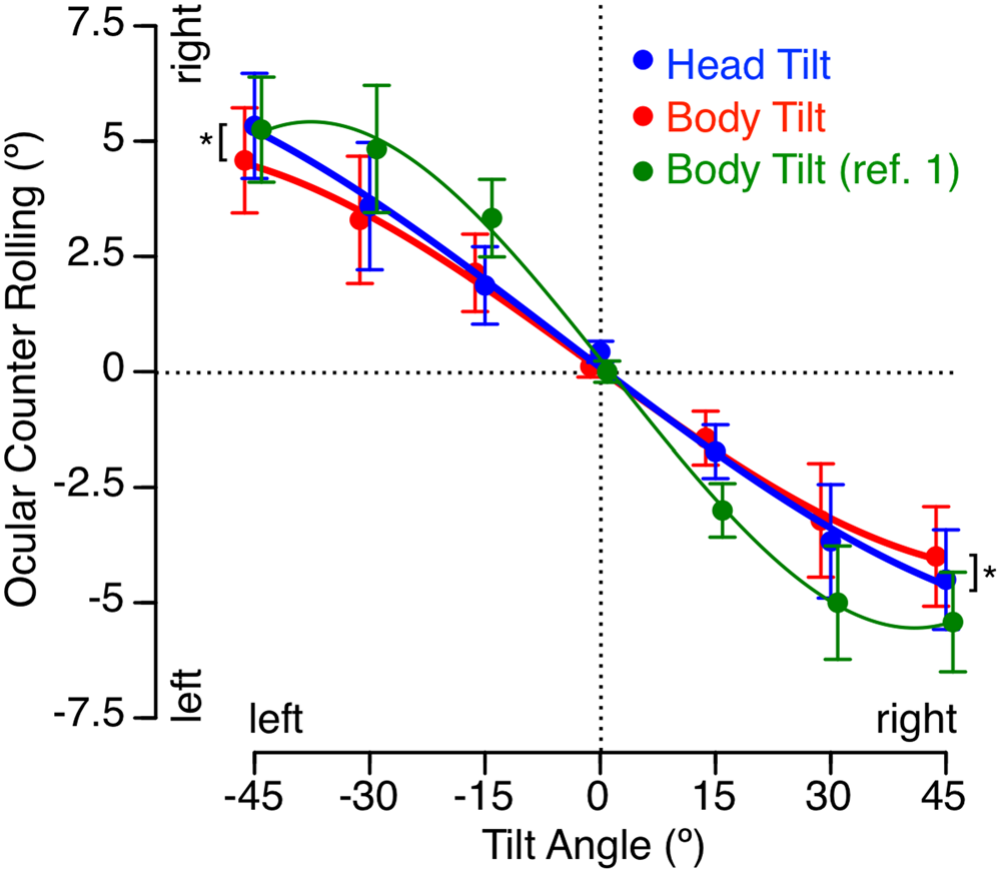
OCR during whole body tilt (in red) and during head tilt (in blue) in the control group (N = 7). Average of the subjects’ three trials at each of the body or head tilt angles. The green symbols/line represent the data from Miller^1^ that were obtained during whole body tilt using photographic methods for measuring eye torsion (N = 100). Second order polynomial least-square curve fitting. Mean ± SD. *P < 0.05 (between whole body tilt and head tilt at ±45°).

The data obtained during both whole body tilt and head tilt showed clear counter-rotation of the eye as a function of utricular shearing force (Fig. 3). A body or head tilt to the right corresponds to torsion of the eye to the left, and vice-versa. A repeated measures ANOVA with 2 factors (tilt angle: 15°, 30°, 45°; direction of tilt: right, left) indicated that there was no statistically significant difference in the OCR during right and left tilts during both whole body tilt [F(2,41) = 1.06, P = 0.31] and head tilt [F(2,41) = 0.79, P = 0.38]. The OCRs were then averaged for both directions of tilt. A repeated measures ANOVA with 2 factors (tilt angle: 0°, 15°, 30°, 45°; tilt conditions: whole body, head) indicated that the OCR significantly increased with the angle of tilt [F(3,55) = 59.6, P < 0.001], and that there was a significant difference between the two tilt conditions [F(1,55) = 4.82, P = 0.03]. Post hoc paired samples t-test indicated that the difference in OCR amplitude between head and body tilt was only significant (P = 0.01) for the largest angle of tilt used (±45°).

The means of OCRs during whole body tilt were not significantly different from the OCRs measured by Miller^1^ using eye photographs [F(1,13) = 0.01, P = 0.91] (Fig. 3). In our study, the asymmetry ratio averaged for all subjects and tilt angles was 19.3% ± 16.7% (mean ± standard deviation) during whole body tilt and 25.3% ± 15.9% during head tilt. This difference in asymmetry was not significant [F(1,41) = 1.5, P = 0.23].

### Space Shuttle study

**Figure 4:**
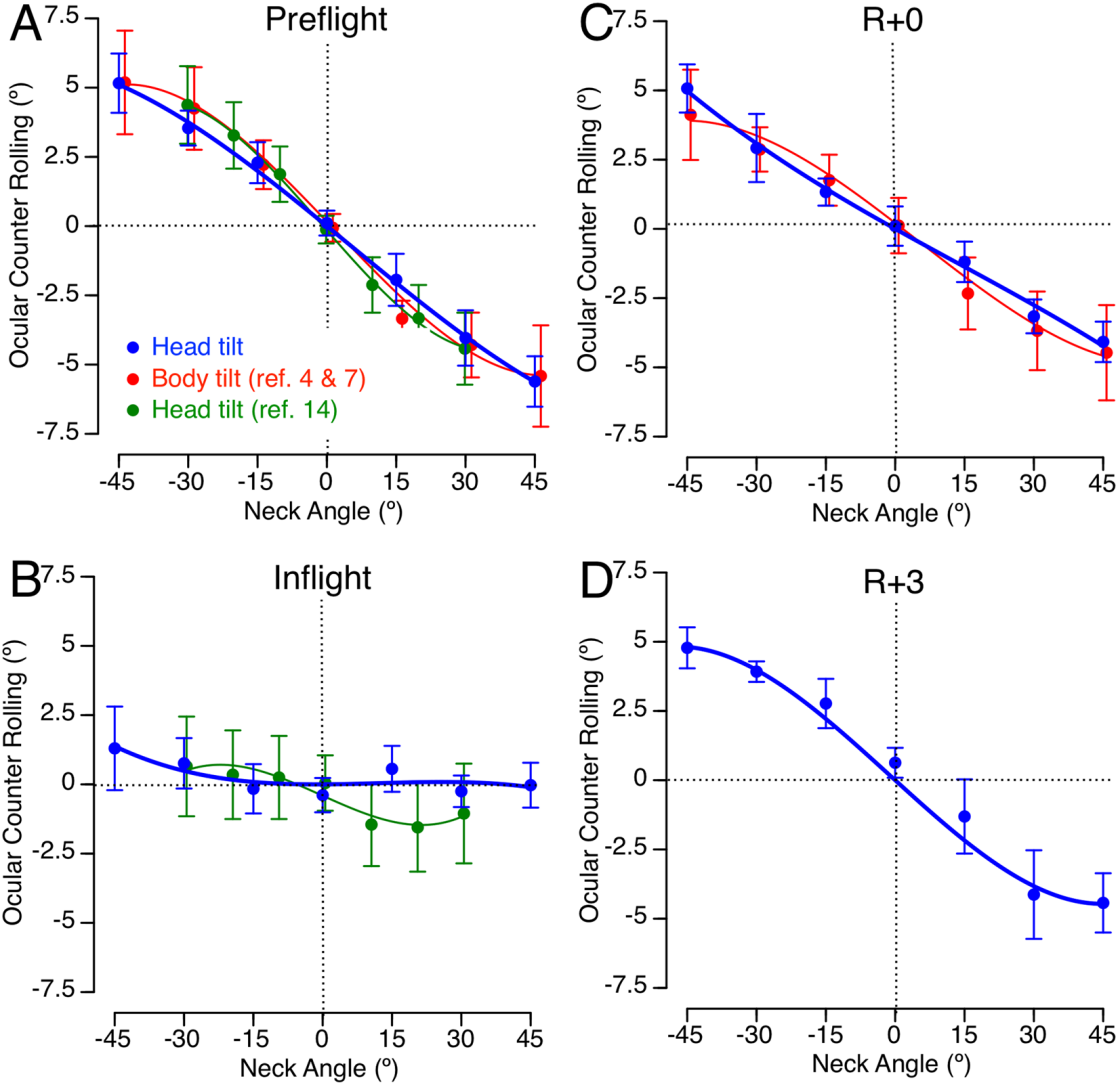
OCR during head tilt relative to the body before (**A**), during **(B**), immediately after (R + 0), and after 3 days (R + 3) after short-duration spaceflight. Blue symbols: Mean ± SD of OCR of the 6 Space Shuttle crewmembers averaged across 3 trials and all 6 preflight (**A**) and inflight (**B**) sessions. Mean ± SD of OCR of each of the 6 crewmembers averaged across 3 trials at each of the neck tilt angles at R + 0 (**C**) and R + 3 (**D**). Second order polynomial least-square curve fitting. Red symbols: Mean ± SD of OCR measured by Vogel & Kass^4^ and Young & Sinha^7^ on 8 crewmembers before and after 2 Space Shuttle missions using photography for measuring eye torsion during whole body tilt. Green symbols: Mean ± SD of OCR measured by Cheung *et al*.^14^ on 6 participants in normal gravity (**A**) and microgravity during parabolic flight (**B**) using eye coils for measuring eye torsion during head tilt.

A repeated measures ANOVA with 2 factors (tilt angle: 0°, 15°, 30°, 45°; preflight session: 2 months, 1 month, 10 days) indicated that there were no statistically significant differences in the OCR of Space Shuttle crewmembers in the 3 preflight sessions [F(2,161) = 2.56, P = 0.08]. There were no significant differences in OCR in the 3 in-flight sessions neither [F(2,161) = 2.13, P = 0.12]. Consequently, the astronauts’ preflight and in-flight OCR measures were averaged for each of the head tilt angles. Figure 4A shows the mean preflight OCR for the 6 participating crewmembers. Each point on the plot represents the average of 3 preflight measurements sessions for each of the 6 crewmembers. Because measures of eye torsion in this study relied on the ability of the subject to align an afterimage placed on the retina with a target template, we compared these results with eye torsion data from 6 non-astronaut subjects that were obtained using scleral coils during head tilt in normal gravity^14^. We also compared these results with video-oculography that was obtained from 8 astronaut-subjects during whole body tilt before spaceflight^4,7^. A single factor ANOVA comparing the mean OCR calculated for the various tilt angles in normal gravity indicated no statistically significant difference between OCR measurements using the afterimage, scleral coils, or video-oculographic methods [F(3,27) = 0.01, P = 0.99].

The mean OCR for all Space Shuttle crewmembers during flight (Fig. 4B) indicates there were no distinct torsional eye movements as a function of head tilt. A repeated measures ANOVA with 2 factors (tilt angle: 0°, 15°, 30°, 45°; session: Pre, In, R + 0, R + 3) indicated a significant effect of tilt angle [F(3,95) = 29.6, P < 0.001] and session [F(3,95) = 18.5, P < 0.001]. Post hoc paired samples t-test corrected for multiple comparisons (Bonferroni) indicated that the OCR amplitude in-flight was significantly different from preflight for all tilt angles (P < 0.001). Two hours after landing, the amplitude of OCR was slightly reduced for all tilt angles compared to preflight levels, but this reduction was not significant (Bonferroni post hoc test; P = 0.34, 0.43, and 0.31 for tilt angles of 15°, 30°, and 45°, respectively) (Fig. 4C). By R + 3, the eye torsion was almost equivalent to preflight measures (Fig. 4D). It is interesting to note that OCR has been reported to dramatically decrease in in microgravity conditions in parabolic flight^14^ (Fig. 4B). Also, the OCR measurements on R + 0 in our study were not significantly different (P = 0.06) from those measured previously in 8 other crewmembers one day after returning from a Space Shuttle mission^4,7^ (Fig. 4C).

**Figure 5:**
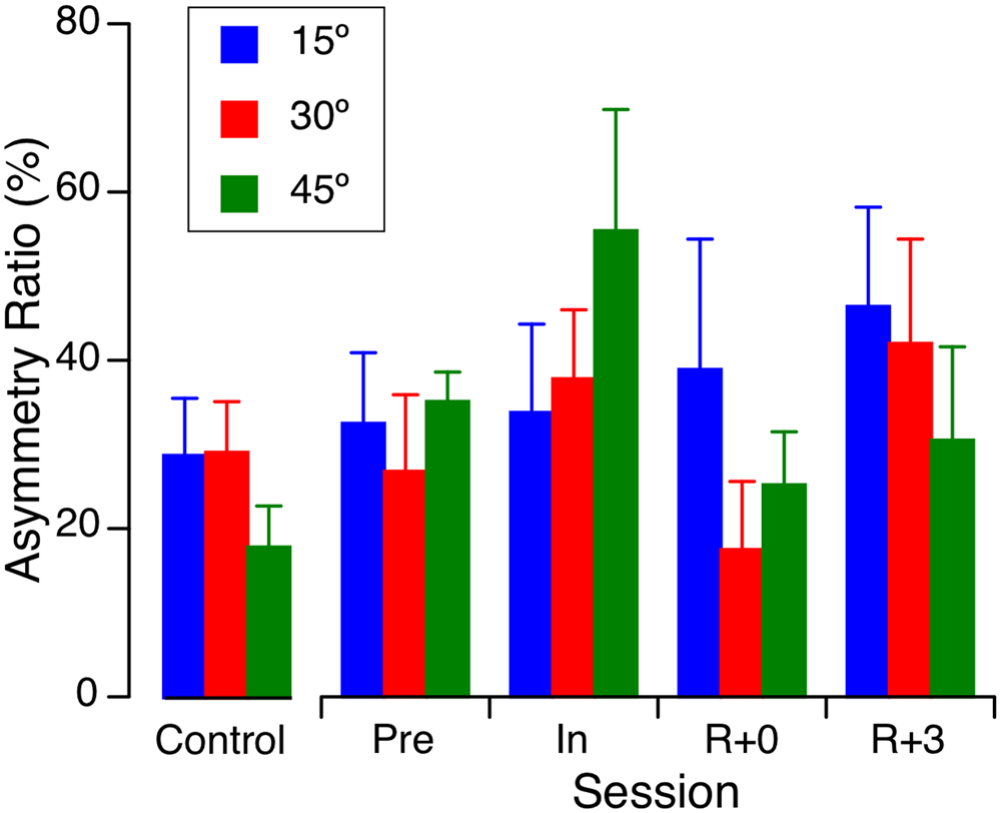
Asymmetry ratio of OCR during head tilt of 15°, 30°, and 45° before (Pre), during (In), and after (R + 0, R + 3) a short-duration spaceflight. Mean ± SD, N = 6. The mean ± SD asymmetry ratio obtained in the control group (N = 7) during head tilt is shown for comparison.

The mean asymmetry ratio before the flight for all 6 Space Shuttle crewmembers and all tilt angles was 31.4% ± 17.5% (mean ± standard deviation), which is comparable to the value obtained in the normative population (Fig. 5). A repeated measures ANOVA with 2 factors (tilt angle: 15°, 30°, 45°; session: Pre, In, R + 0, R + 3) indicated no significant effect of tilt angle [F(2,71) = 0.49, P = 0.61] and session [F(3,71] = 1.36, P = 0.26].

### International Space Station study

**Figure 6:**
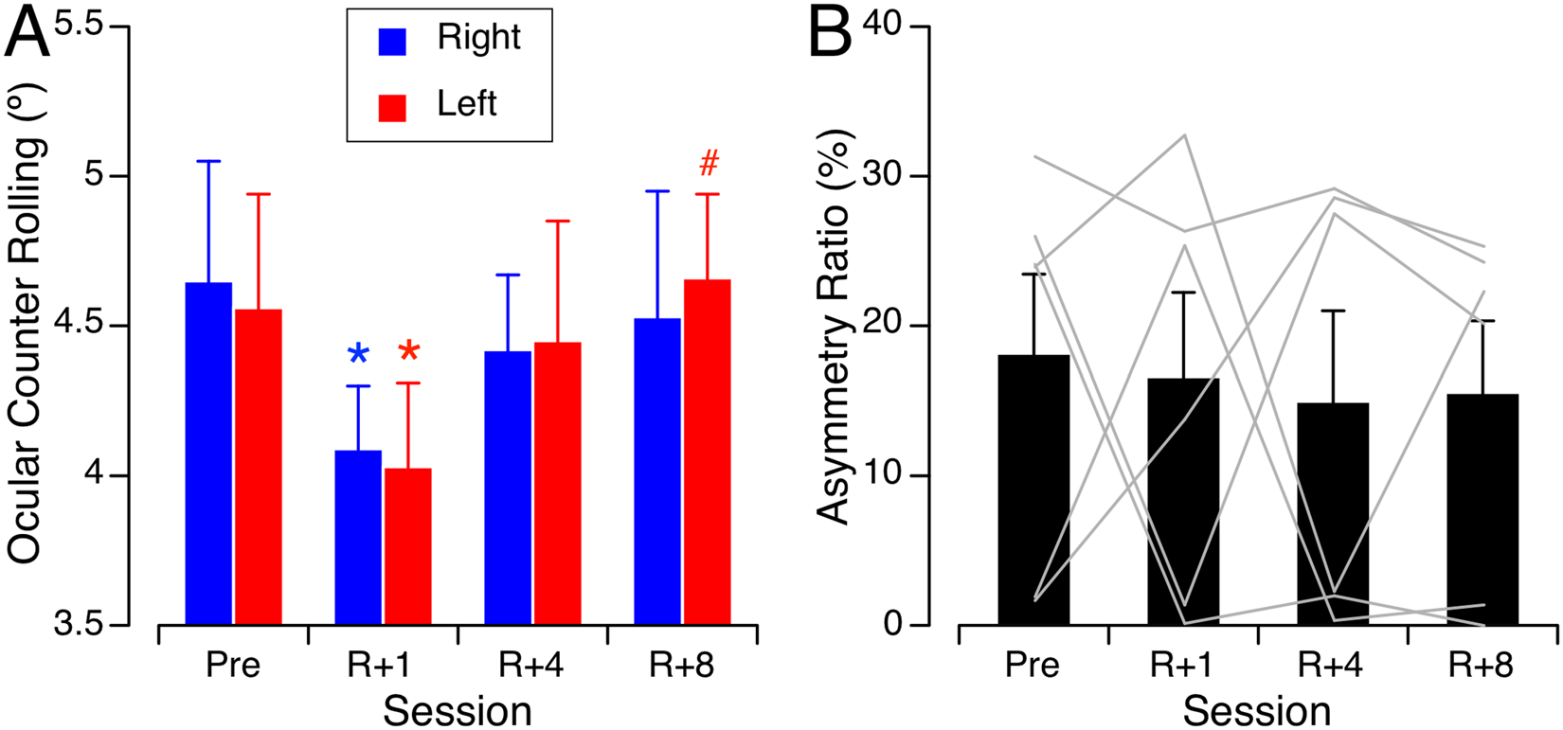
(**A**) Right and left OCR during body tilt of ±25° relative to gravity before and after a long-duration spaceflight. Mean ± SD of the 6 ISS crewmembers averaged across all 3 preflight sessions (Pre) and during each post-flight session. *P < 0.05 relative to preflight; ^#^P < 0.05 relative to R + 1. (**B**) Asymmetry ratio of OCR of the 6 ISS crewmembers. Mean ± SD. Gray lines show individual data.

A single factor ANOVA indicated that there were no significant differences in the OCR amplitudes for the 6 ISS crewmembers during the 3 preflight sessions [F(2,17) = 0.01, P = 0.99] for 25° tilt to the right; F(2,17) = 1.08, P = 0.36 for 25° tilt to the left), so the values were averaged. The mean OCR amplitude decreased significantly on R + 0 compared to preflight values [F(3,23) = 3.98, P = 0.02], and returned to baseline by R + 4 (Fig. 6A). The mean asymmetry ratio for all 6 ISS crewmembers was 18.2% ± 12.9% before the flight; it did not change significantly after the flight [F(3,23) = 0.06, P = 0.97] (Fig. 6B).

## Discussion

The results of this study indicate that OCR during head tilt is absent in microgravity and returns to normal within 2 hours of return from a short-duration spaceflight. However, after a long-duration spaceflight, the OCR amplitude is reduced relative to preflight for approximately 36 hours after landing and has returned to normal 4 days after landing. This decrease in amplitude is not accompanied by changes in the asymmetry of OCR between right and left head tilt. This result suggests that the ocular otolith-driven reflexes take more than a few days to adapt to microgravity.

### Comparison of OCR during whole body tilt and head tilt

Our OCR measures obtained during whole body tilt on Earth are comparable to the OCR measures reported in previous studies using photographic^15,16^ and video-oculographic^4,7,11^ methods to measure eye torsion. Our measurements of OCR during head tilt on Earth are also similar to the results of previous studies using the scleral coils technique^14,17,18^.

Larger OCR amplitudes were measured during head tilts at 45° compared to whole body tilt at 45°. It is possible that the goggles may have slipped relative to the head at this more extreme angle of tilt, which would account for this effect. Another possible interpretation is that somatosensory or cervical inputs contribute to eye torsion in an additive fashion at larger angles, a process normally attributed to otolith shearing forces only. The evidence for the contribution of somatosensory or cervical inputs comes from studies that show residual OCR in labyrinthine-defective subjects^17,19^.

### Effects of microgravity on OCR

Previous studies using head tilt have shown that OCR amplitude was smaller in microgravity and higher in hypergravity during parabolic flight compared to amplitudes in normal gravity^14,20,21^. Hoffstetter-Degen *et al*.^5^ observed that static OCR did not occurred in one astronaut when he rolled his head onto his shoulder on days 3, 5, and 7 of his spaceflight. Our results confirm this observation and indicate that no neck receptor mediated component exists in static OCR in microgravity. When the head is tilted in microgravity, no linear acceleration is exerted on the utricles and OCR is absent. In the same tilted position under hypergravity, greater linear acceleration is exerted on the utricles and more ocular torsion is induced.

**Figure 7:**
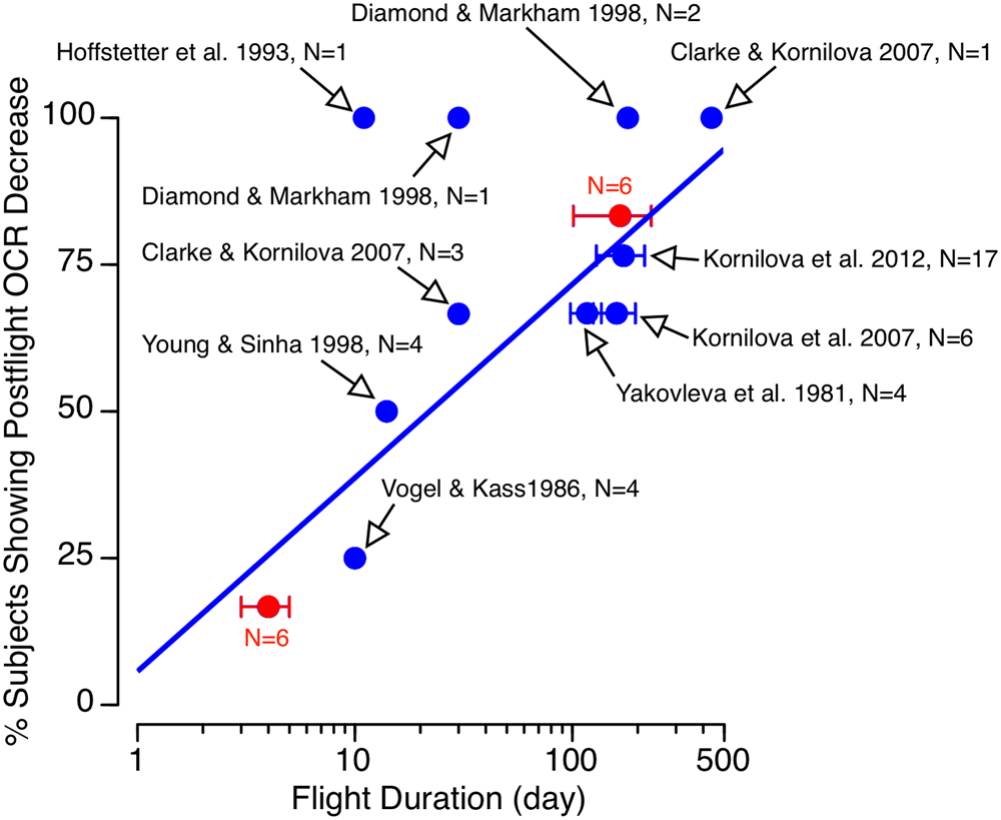
Overall results of 11 OCR studies performed before and after spaceflight. Each data point represents the percentage of subjects showing an OCR decrease postflight relative to preflight for the duration of the flight corresponding to that particular study. Study reference and number of subjects tested are indicated next to each data point. Results of the present Space Shuttle and ISS studies are shown in red. Note the logarithmic scale on the x-axis (Flight Duration). The blue line depicts a logarithmic function fitted to the data (r^2^ = 0.69).

Our results show no postflight changes in OCR after 4–6 days Space Shuttle flights and a postflight decrease in OCR after 4–9 months ISS flights relative to preflight values, which suggests that the duration of exposure to microgravity is a critical factor in the adaptation of this otolith-mediated reflex. Distinct decreases in static OCR have been measured in cosmonauts for several days following long-duration stays (3–14 months) on board the Mir space station^9–11,22^. However, inter-individual differences were present after Soyuz and Space Shuttle missions lasting 3–15 days; some subjects’ OCR increased after flight relative to baseline, whereas others’ OCR decreased after flight^4,7,8^. Figure 7 shows the percentage of subjects that demonstrated a significant OCR decrease after spaceflight compared to preflight in each of these studies. The results from the present Space Shuttle and ISS studies are also included. The longer the duration of the spaceflight, the higher percentage of subjects showing a postflight OCR decrease, thus indicating that the adaptation of this otolith-mediated reflex takes place throughout the flight.

A decrease in static OCR after return from long-duration spaceflight is consistent with the hypothesis that central compensatory mechanisms are activated during prolonged exposure to microgravity for adjusting otolith-mediated responses. On Earth, information from the otolith receptors is interpreted as either linear motion or as head or body tilt with respect to gravity. Because stimulation from gravity is absent during spaceflight, interpretation of otolith input as tilt is meaningless. After several weeks in space, the brain adapts to weightlessness by reinterpreting all otolith receptor output as linear acceleration, and stimulation of the otoliths is interpreted as translation. Immediately after returning from a long spaceflight, and before the CNS readapts to the normal gravity force environment, this new interpretation persists, during which linear acceleration and tilt are both perceived as translation^2,3^. Consequently, during this post-flight period the otolith-mediated tilt responses, such as the static OCR, are reduced.

Eccentric rotation about the vertical axis, translation along the horizontal (inter-aural) axis, and off-vertical axis rotation also generate linear acceleration along the utricular macula, which in turn induces OCR. In agreement with our results, the amplitude of OCR induced by eccentric rotation about the vertical axis was the same as preflight in 4 astronauts after short (16-day) spaceflights^23^, but significantly decreased in 34 astronauts after long (6-month) spaceflight^24^ compared to baseline. Also, the OCR amplitude induced by off-vertical axis rotation was not different from preflight in 8 astronauts after short Space Shuttle missions^25^. The OCR in response to sinusoidal linear translation^26,27^ and unilateral centrifugation along the inter-aural axis^28^ after short spaceflights decreased from preflight values in some subjects and increased in others.

### Otolith asymmetry

Deconditioning of otolith-mediated tilt responses after adaptation to microgravity has been proposed as the basis of many of the perceptual, postural, locomotor, and gaze control problems that astronauts experience on return to Earth^2^. Von Baumgarten and Thumler^29^ suggested that a slight innate bilateral asymmetry exists in the otoconial mass of the otolith organs. In normal gravity, inputs from visual and somatosensory receptors centrally compensate for this anatomical difference by constantly fine-tuning the otolith information to maintain the appropriate torsional eye position. In microgravity, the persisting central compensation would produce an inappropriate signal, which manifests in some subjects as inappropriate ocular, perceptual, and neurovegetative responses^30^.

During in-orbit centrifugation along the inter-aural axis, one crewmember developed an OCR asymmetry that persisted for 9 days after return from a Space Shuttle mission^23^. Asymmetries in OCR in response to sinusoidal body translation have also been observed in Space Shuttle astronauts after flight^4^. However, in agreement with our results, no significant asymmetry in the OCR response to static head tilt was observed in cosmonauts after long-duration spaceflight^11^. Our results therefore do not support the hypothesis of an asymmetry between labyrinths that is compensated during spaceflight.

Although static OCR in response to head tilts is consistently in the compensatory direction, its gain never exceeds 20%, so OCR does not play a major role in spatial orientation^31^. Crewmembers experience spatial disorientation episodes after both short- and long-duration space missions^32^. Our results indicate that changes in OCR gain are mostly significant after long-duration spaceflight, so the contribution of the OCR to spatial disorientation is questionable. However, decreases in OCR after a long-duration exposure to microgravity could also be due to mechanical alterations of the otolith organs, such as a change in their mass caused by a loss of calcium in microgravity. Morphological and neurophysiological studies of animals that have flown in space have shown that spaceflight changes the structure of the otoconia^33^, increases the number of synapses in utricular macular hair cells, and changes the sensitivity of utricular afferents^34,35^. To date, the neurovestibular symptoms in astronauts are only crudely evaluated using a questionnaire 3 days after return to Earth. Now that the space agencies have plans for deep space exploration missions that will last up to 3 years, more complete evaluations of neurovestibular function are needed in orbit and immediately after long-duration spaceflight, to demonstrate whether changes are taking place in the otoconia, the peripheral neural network, and/or the central vestibular system.
